# Characterization of high- and low-risk hepatocellular adenomas by magnetic resonance imaging in an animal model of glycogen storage disease type 1A

**DOI:** 10.1242/dmm.038026

**Published:** 2019-04-05

**Authors:** Roberta Resaz, Francesca Rosa, Federica Grillo, Luca Basso, Daniela Segalerba, Andrea Puglisi, Maria Carla Bosco, Luca Mastracci, Carlo E. Neumaier, Luigi Varesio, Alessandra Eva

**Affiliations:** 1 Laboratory of Molecular Biology, Istituto Giannina Gaslini, 16147 Genova, Italy; 2 Department of Science of Health (DISSAL), University of Genova, 16132 Genova, Italy; 3 Department of Radiology, Ospedale Policlinico San Martino, 16132 Genova, Italy; 4 Pathology Unit, Department of Surgical Sciences and Integrated Diagnostics (DISC), University of Genova, 16132 Genova, Italy; 5 Anatomic Pathology, Ospedale Policlinico San Martino, 16132 Genova, Italy

**Keywords:** Liver, Tumor, Magnetic resonance, β-Catenin

## Abstract

Hepatocellular adenomas (HCAs) are benign tumors, of which the most serious complications are hemorrhage and malignant transformation to hepatocellular carcinoma (HCC). Among the various subtypes of HCA, the β-catenin-activated subtype (bHCA) is associated with greatest risk of malignant transformation. Magnetic resonance imaging (MRI) is an important tool to differentiate benign and malignant hepatic lesions, and preclinical experimental approaches may help to develop a method to identify MRI features associated with bHCA. HCAs are associated with various pathologies, including glycogen storage disease 1a (GSD1a). Here, we utilized a mouse model for GSD1a that develops HCA and HCC, and analyzed the mice in order to distinguish low-risk from high-risk tumors. Animals were scanned by MRI using a hepato-specific contrast agent. The mice were sacrificed after MRI and their lesions were classified using immunohistochemistry. We observed that 45% of the animals developed focal lesions, and MRI identified four different patterns after contrast administration: isointense, hyperintense and hypointense lesions, and lesions with peripheral contrast enhancement. After contrast administration, only bHCA and HCC were hypointense in T1-weighted imaging and mildly hyperintense in T2-weighted imaging. Thus, high-risk adenomas display MRI features clearly distinguishable from those exhibited by low-risk adenomas, indicating that MRI is a reliable method for early diagnosis and classification of HCA, necessary for correct patient management.

## Abstract (precis)

**Summary:** High-risk adenomas display magnetic resonance imaging (MRI) features clearly distinguishable from those exhibited by low-risk adenomas, suggesting the possibility of applying MRI to humans for non-invasive diagnostic purposes.

## INTRODUCTION

Hepatocellular adenomas (HCAs) are benign tumors that constitute 2% of all liver tumors, and their most important complications are hemorrhage and malignant transformation to hepatocellular carcinoma (HCC) (Stoot et al., 2010).

HCAs have been classified into four subgroups on the basis of their pathogenesis: HHCA, containing bi-allelic inactivating mutations of hepatocyte nuclear factor 1 alpha; bHCA, characterized by activating mutations of the gene encoding β-catenin and associated with greater risk of malignant transformation than the other subtypes; inflammatory HCA (IHCA), characterized by inflammatory infiltrates and high expression of C-reactive protein (CRP) and serum amyloid A2 (SAA2) (∼10% of IHCAs are also β-catenin mutated); and unclassified HCA (UHCA), accounting for less than 10% of the cases (Bioulac-Sage et al., 2010, 2007; Zucman-Rossi et al., 2006; Nault et al., 2017).

HCAs are frequently associated with glycogen storage disease (GSD) 1a (Franco et al., 2005; Labrune et al., 1997; Reddy et al., 2007). GSD1a is an autosomal recessive disorder caused by mutations in the catalytic subunit of glucose-6-phosphatase-alpha (G6Pase-α), a key enzyme in glucose metabolism (Lei et al., 1993). The chronic dysmetabolism affects the functions of liver and kidney, resulting in a progressive worsening of the clinical parameters and development of hepatic adenomas that may undergo malignant transformation. In a European cohort study, HCAs were observed in 80% of the patients with GSD1a above the age of 30 years. Furthermore, 10% of GSD patients developed HCC (Lee, 2002).

Fifty-two percent of GSD adenomas can be classified as IHCA, 28% as bHCA and the remaining 20% as UHCA (Calderaro et al., 2013). The elevated percentage of adenomas with β-catenin-activating mutations may explain the risk of malignant transformation of HCA in HCC in GSD1a patients.

Magnetic resonance imaging (MRI) represents an important tool to differentiate benign and malignant hepatic lesions, particularly because of the possibility to combine information obtained with different sequences, such as T1- and T2-weighted imaging (WI), diffusion WI (DWI) and post-contrast T1-WI, using hepatobiliary gadolinium contrast agents such as gadobenate dimeglumine (Gd-BOPTA) and gadoxetic acid (Gd-EOB) (Oliva and Saini, 2004).

Correlation between MRI and the genotype-phenotype classification of HCAs has been attempted (Laumonier et al., 2008; van Aalten et al., 2011; Yoneda et al., 2012). Characteristic MRI profiles for HHCA and IHCA have been identified, but no specific MRI profile can be proposed for the identification of high-risk bHCA cases. In consideration of this, the subtyping and classification of liver adenomas for HCC risk prediction in humans still requires invasive procedures, i.e. biopsies.

A preclinical experimental approach may help to develop a method to better identify MRI features specifically associated with bHCA subtype. In this respect, we have generated and characterized a mouse model for GSD1a in which the deletion of *G6pc*, the gene encoding G6Pase-α, occurs only in the liver. These mice (LS-*G6pc*^−/−^) mimic all the steps of the liver disease progression, including the development of HCA and transformation to HCC, providing a link between G6Pase-α deficiency and neoplastic liver progression (Resaz et al., 2014).

In this study, we assessed the detection level and quantified the longitudinal evolution of liver lesions in LS-*G6pc*^−/−^ mice in order to characterize HCAs and HCC using a dedicated protocol, optimized for high magnetic field strength, and a hepato-specific contrast agent. We show that bHCA displays MRI features distinguishable from those exhibited by IHCA or UHCA, indicating that MRI is a reliable method to identify high-risk HCA.

## RESULTS

### Characterization of hepatic focal lesions in LS-*G6pc*^−/−^ mice

**Fig. 1.**
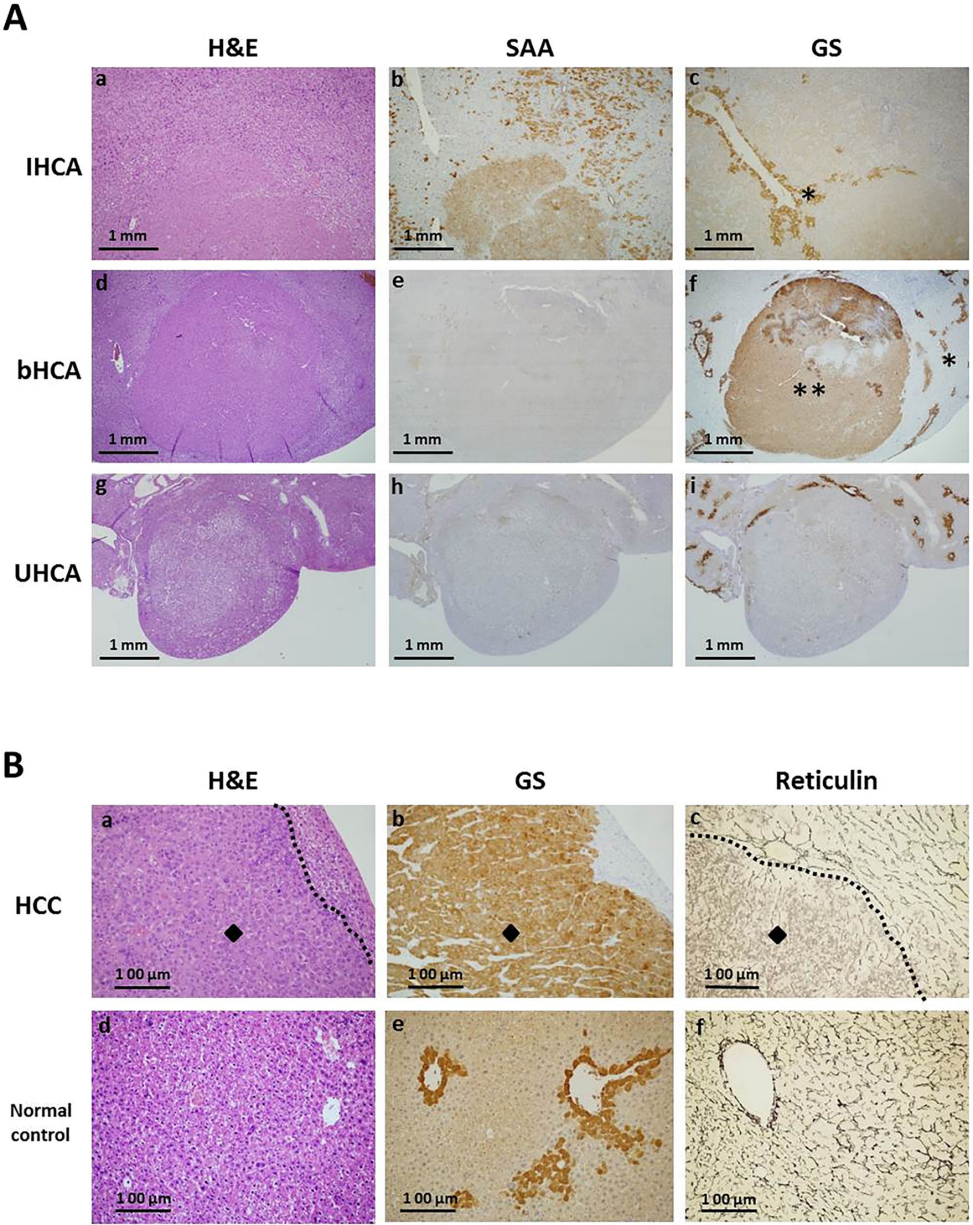
**Immunoclassification of adenomas in LS-*G6pc*^−/−^ mice.** LS-*G6pc*^−/−^ mice livers were fixed with 10% formalin and paraffin embedded. Serial sections were stained with Hematoxylin and Eosin (H&E) or treated with anti-serum amyloid (SAA) or anti-glutamine synthetase (GS) antibody. (A) Histological features and immunohistochemistry analysis of liver adenomas in LS-*G6pc*^−/−^ mice. H&E-stained section of an IHCA (a). An IHCA nodule shows diffuse positivity for SAA within the lesion, while the surrounding parenchyma is negative except for perivascular amyloid deposition (b). The same nodule shows negativity for GS antibody, while the surrounding non-lesional parenchyma shows normal GS staining in perivascular hepatocytes (asterisk) (c). The images shown are representative of 17 IHCA lesions examined. H&E-stained section of a bHCA (d). A bHCA nodule is negative for SAA (e) but shows strong positivity for GS within the lesion (double asterisk), while the surrounding non-lesional parenchyma shows normal zonation of expression in perivascular hepatocytes (asterisk) (f). The images shown are representative of eight bHCA lesions examined. H&E-stained section of an UHCA (g). An UHCA nodule is negative for both SAA (h) and GS (i). The images shown are representative of 12 UHCA lesions examined. (a-c) 10× magnification; (d-i) 4× magnification. (B) Histological features and immunoexpression of GS in a hepatocellular carcinoma (diamonds) (a,b,c) and in a normal control murine liver (d,e,f). (a) H&E-stained section of a nodule showing atypia (diamond); a contour (dotted line) has been drawn between the HCC (left) and normal surrounding tissue (right). (b) Strong and diffuse positivity to GS within the lesion (diamond). (c) An HCC section with Gomori staining for reticulin framework, showing loss of reticulin framework typical of malignancy (diamond). Reticulin is preserved in the surrounding parenchyma; a contour (dotted line) has been drawn between the HCC (left) and normal surrounding tissue (right). The images shown are representative of three HCCs examined. (d) H&E-stained section of normal liver. (e) Strong positivity to GS is observed in the perivascular hepatocytes of a normal liver control. (f) Gomori staining of normal liver showing preserved reticulin trabecular framework and perivascular collagen. (a-f) 20× magnification.

LS-*G6pc*^−/−^ mice livers were histologically examined. Liver parenchyma showed the classical histological features of GSD1a (Resaz et al., 2014; Chou et al., 2010). All HCAs were histologically examined and immunostained for pathological classification. Histological analysis of HCA of LS-*G6pc*^−/−^ mice showed that they were unencapsulated nodules composed of hepatocytes with no or little atypia and with no or few intralesional portal tracts (Fig. 1A,a,d,g).

Anti-glutamine synthetase (GS) antibody, to identify bHCA (high-risk adenoma), and anti-serum amyloid A (SAA) antibody, to identify IHCA, was used. For GS, only a strong and diffuse cytoplasmic staining was considered positive, and staining intensity was comparable to the staining in normal liver in the centrilobular, perivenular hepatocytes. SAA marked IHCA hepatocytes with granular cytoplasmic staining and amyloid deposits in vascular and/or sinusoidal spaces. We previously reported that 90% of LS-*G6pc*^−/−^ mice develop amyloidosis by 12 months of age (Resaz et al., 2014).

HNF1α-mutated HCAs have not been identified in GSD-related HCAs (Calderaro et al., 2013). Therefore, silenced FABP1 was not searched for in the LS-*G6pc*^−/−^ mouse liver tumors.

Eighteen nodules stained positive for SAA and negative for GS, and were identified as IHCA (Fig. 1A,b,c); eight nodules stained negative for SAA and positive for GS, and were identified as high-risk bHCA (Fig. 1A,e,f); and 12 nodules were negative for both SAA and GS, and were classified as UHCA (Fig. 1A,h,i). Moreover, three lesions were well-differentiated HCCs (Fig. 1B,a), characterized by positive staining for GS (Fig. 1B,b) and loss of reticulin framework (Fig. 1B,c). These results, summarized in Table 1, are in agreement with the molecular characterization of HCA in patients with GSD1a (Calderaro et al., 2013).

**Table 1.**
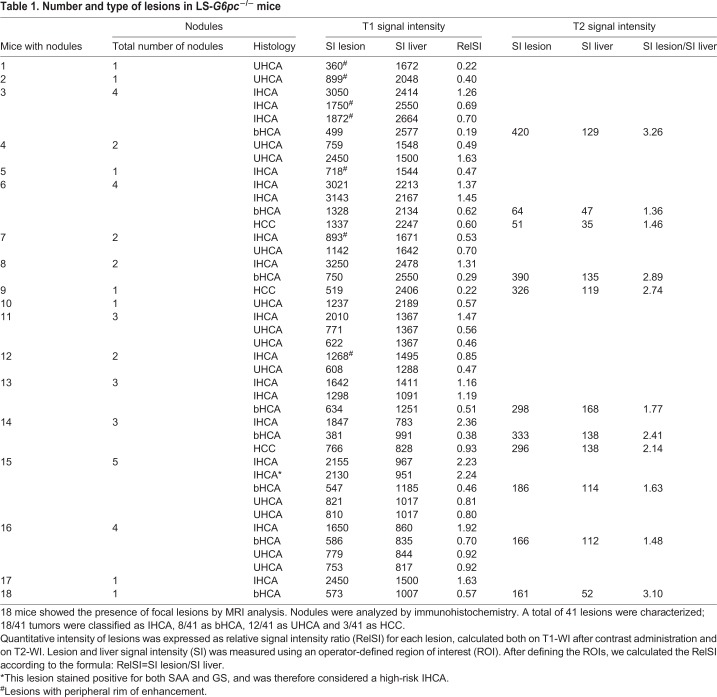
**Number and type of lesions in LS-*G6pc*^−/−^ mice**

### Correlation between MRI signals and immunohistological analysis in livers of LS-*G6pc*^−/−^ mice

To characterize the hepatic lesions detected in LS-*G6pc*^−/−^ mice and correlate MRI features with the different adenoma subtypes, 40 animals aged 13-18 months were scanned by 3T clinical MRI and checked for focal lesions in all sequences acquired. A total of 41 liver lesions were detected in 18 of the 40 animals analyzed, with an incidence of 45%. Twenty-five mice were scanned once and sacrificed immediately after MRI acquisition. The remaining 15 mice were monitored every 4 weeks for 3 months to evaluate lesion growth and complication onset, such as rupture and bleeding (see Table 2), as described below.

**Table 2.**
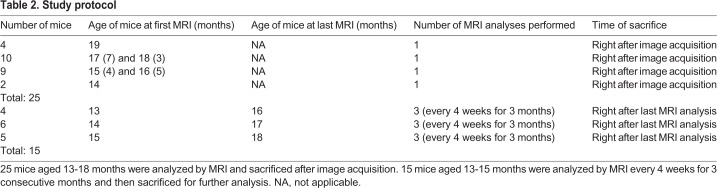
**Study protocol**

**Fig. 2.**
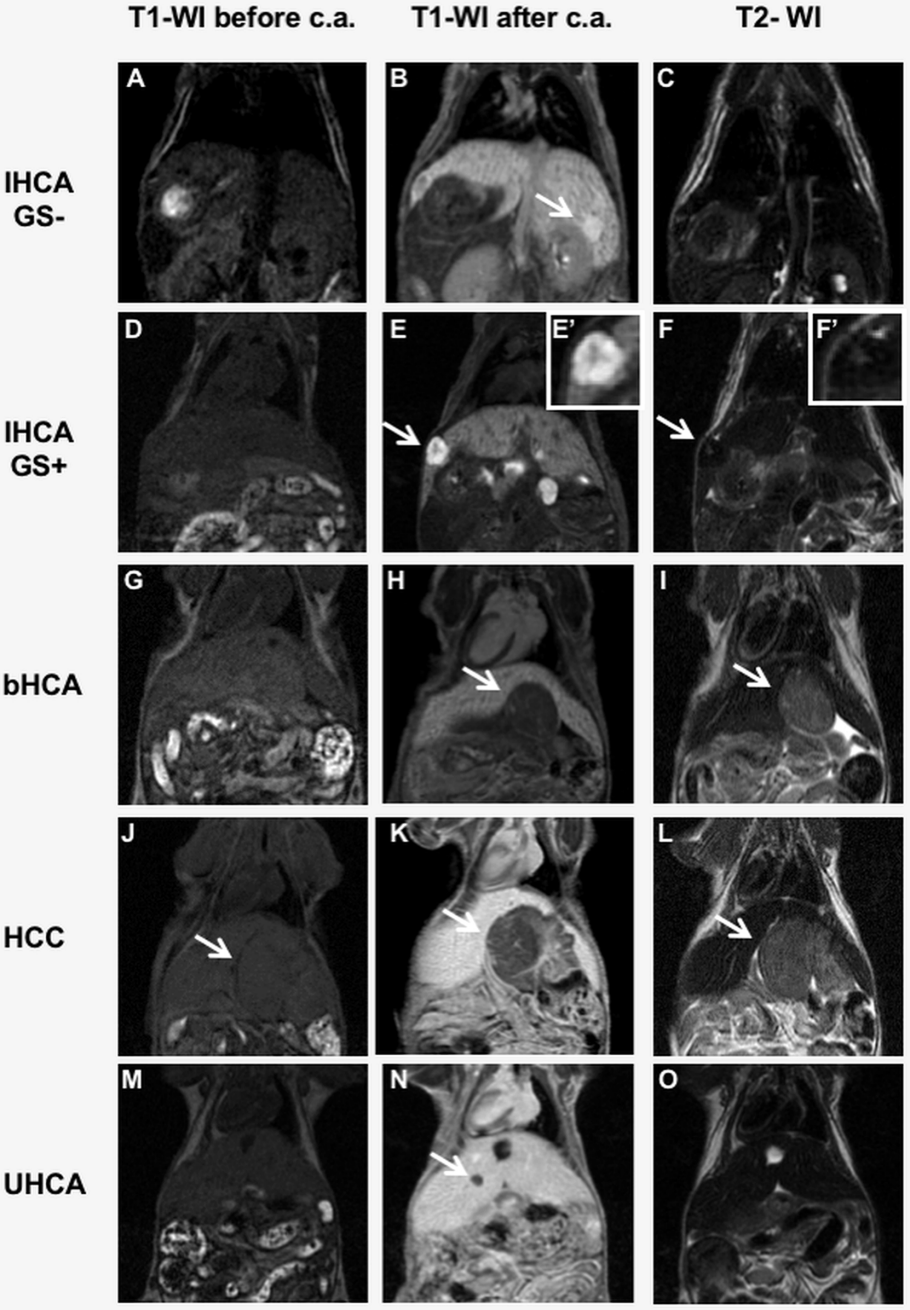
**MRI of various subtypes of liver lesions in LS-*G6pc*^−/−^ mice.** LS-*G6pc*^−/−^ mice were scanned by MRI and checked for focal lesions in all acquired sequences. (A-C) A typical IHCA is isointense on T1-WI before contrast administration (c.a.) (A) and on T2-WI (C), while on T1-WI after c.a. it shows areas of hyperintensity compared to the surrounding liver (B, arrow). The images shown are representative of 17 IHCA lesions examined. (D-F) The single atypical IHCA is isointense on T1-WI before c.a. (D), hyperintense on T1-WI after c.a. (E, arrow) and isointense on T2-WI (F), and characterized by the presence of a faint hypointense central scar (E’) that is mildly hyperintense on T2-WI (F, arrow and F′). (G-I). A bHCA lesion is isointense compared to the liver parenchyma on T1-WI before c.a. (G), hypointense on contrast-enhanced T1-WI (H, arrow) and mildly hyperintense on T2-WI (I, arrow). The images shown are representative of eight bHCA lesions examined. (J-L) The HCC shown is isointense on T1-WI acquired before c.a. (J, arrow), while it shows prominent hypointensity on T1-WI after c.a. (K, arrow) and is slightly hyperintense on T2-WI (L, arrow). The images shown are representative of three HCCs examined. (M-O) A UHCA lesion is isointense on T1-WI before c.a. (M) and hypointense on T1-WI after c.a. (N, arrow). The nodule is isointense compared to the liver parenchyma on T2-WI, different from bHCA and HCC (O). The images shown are representative of 12 UHCAs examined.

**Fig. 3.**
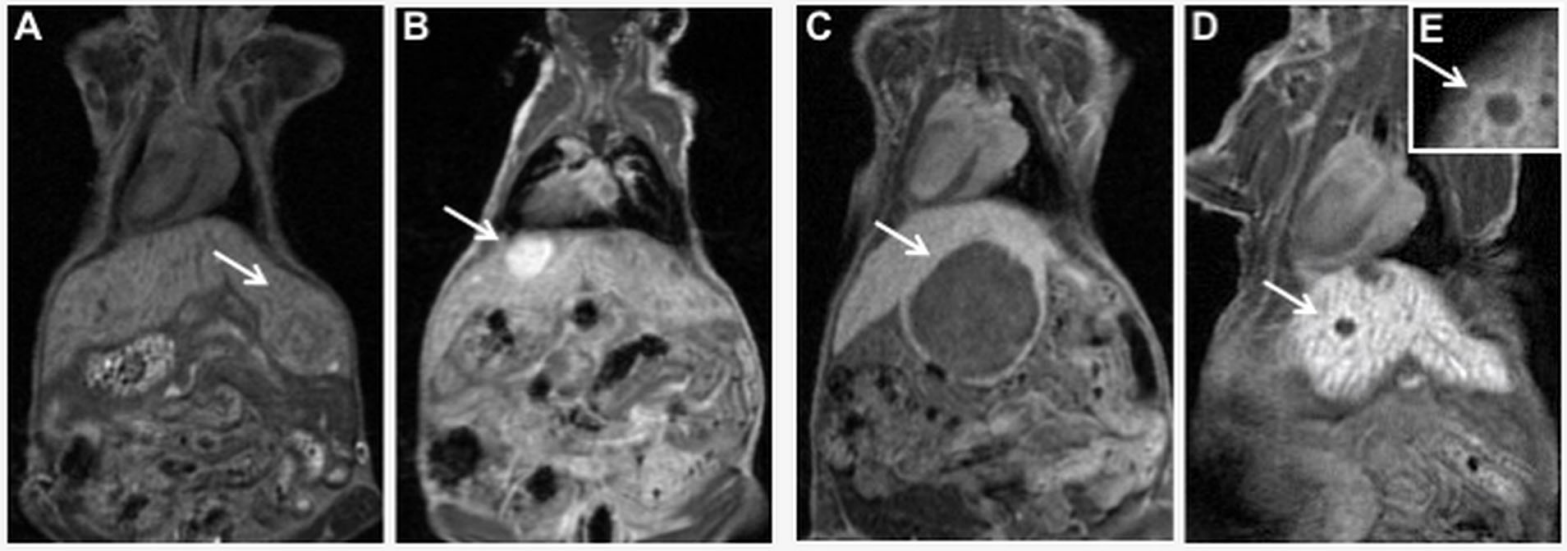
**Different enhancement patterns of focal hepatic lesions.** LS-*G6pc*^−/−^ mice were scanned by MRI. (A-E) The four different patterns of contrast uptake are shown: isointense (A); hyperintense (B); hypointense (C); hypointense lesions with peripheral rim of contrast enhancement from two different mice (D,E). Lesions are indicated by arrows. The results shown are representative of 40 mice examined.

MRI identified four different patterns of contrast uptake after contrast administration: isointense, very hyperintense, and very hypointense lesions, and lesions with a peripheral contrast enhancement. All typical IHCA lesions were isointense on pre-contrast T1-WI and on T2-WI (Fig. 2A,C). On T1-WI after contrast administration, 11/18 (61%) of the IHCA lesions showed a very hyperintense signal compared to the surrounding liver (Fig. 2B), while 6/18 (33%) showed a peripheral rim of contrast enhancement and central hypointensity (Fig. 3D,E). We observed one case of adenoma (1/18, 6%) featuring high expression of both SAA and GS (data not shown). This lesion was isointense on pre-contrast T1-WI (Fig. 2D) and, after contrast administration, was hyperintense and characterized by the presence of a faint hypointense central scar (Fig. 2E,E′). On T2-WI, the lesion was isointense, while the central scar was mildly hyperintense (Fig. 2F,F′).

None of the bHCA lesions, featuring GS overexpression, showed hyperintensity compared to the surrounding liver in the hepatocyte phase or peripheral rim of contrast enhancement after contrast sequences. We observed that 8/8 lesions (100%) were very hypointense after contrast administration on T1-WI and mildly hyperintense on T2-WI (Fig. 2G-I).

All well-differentiated HCCs (3/3) were mildly hyperintense on T2-WI, isointense on T1-WI before contrast, and very hypointense (2/3) (Fig. 2J-L) or mildly hypo/isointense (1/3) on T1-WI after contrast administration (not shown).

UHCA showed all four different contrast enhancement patterns on T1-WI after contrast: the majority (7/12) were very hypointense lesions, 2/12 were isointense, 1/12 were very hyperintense and 2/12 were with a peripheral ring of contrast enhancement. Fig. 2M-O show representative images of a very hypointense UHCA lesion. All UHCA lesions were isointense on T2-WI (Fig. 2O), unlike bHCA and HCC lesions, which were mildly hyperintense (Fig. 2I,L).

These results, summarized in Table 3, indicate that our MRI protocol can be used to identify HCA and to distinguish high-risk from low-risk adenomas. MRI could therefore represent a less-invasive tool for early diagnosis and classification of HCA necessary for correct patient management.

**Table 3.**
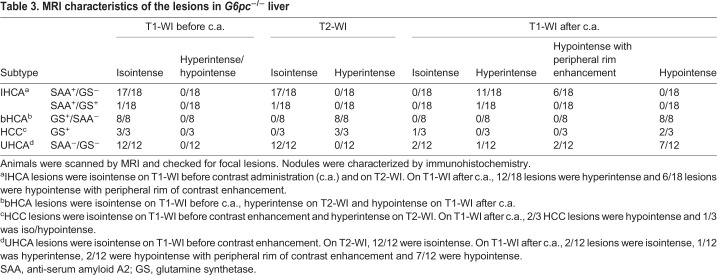
**MRI characteristics of the lesions in *G6pc*^−/−^ liver**

### Monitoring of hepatic lesions

In order to characterize the evolution and growth of the liver lesions in LS-*G6pc*^−/−^ mice, we performed periodic MRI on 15 LS-*G6pc*^−/−^ mice aged 13-15 months. The animals were monitored every 4 weeks for 3 months, and the tumor growth was analyzed by measuring lesion diameter using the off-line workstation. Tumor measurement was performed on the basis of T1-weighted contrast-enhanced scans. Seven mice showed the presence of nodules.

**Fig. 4.**
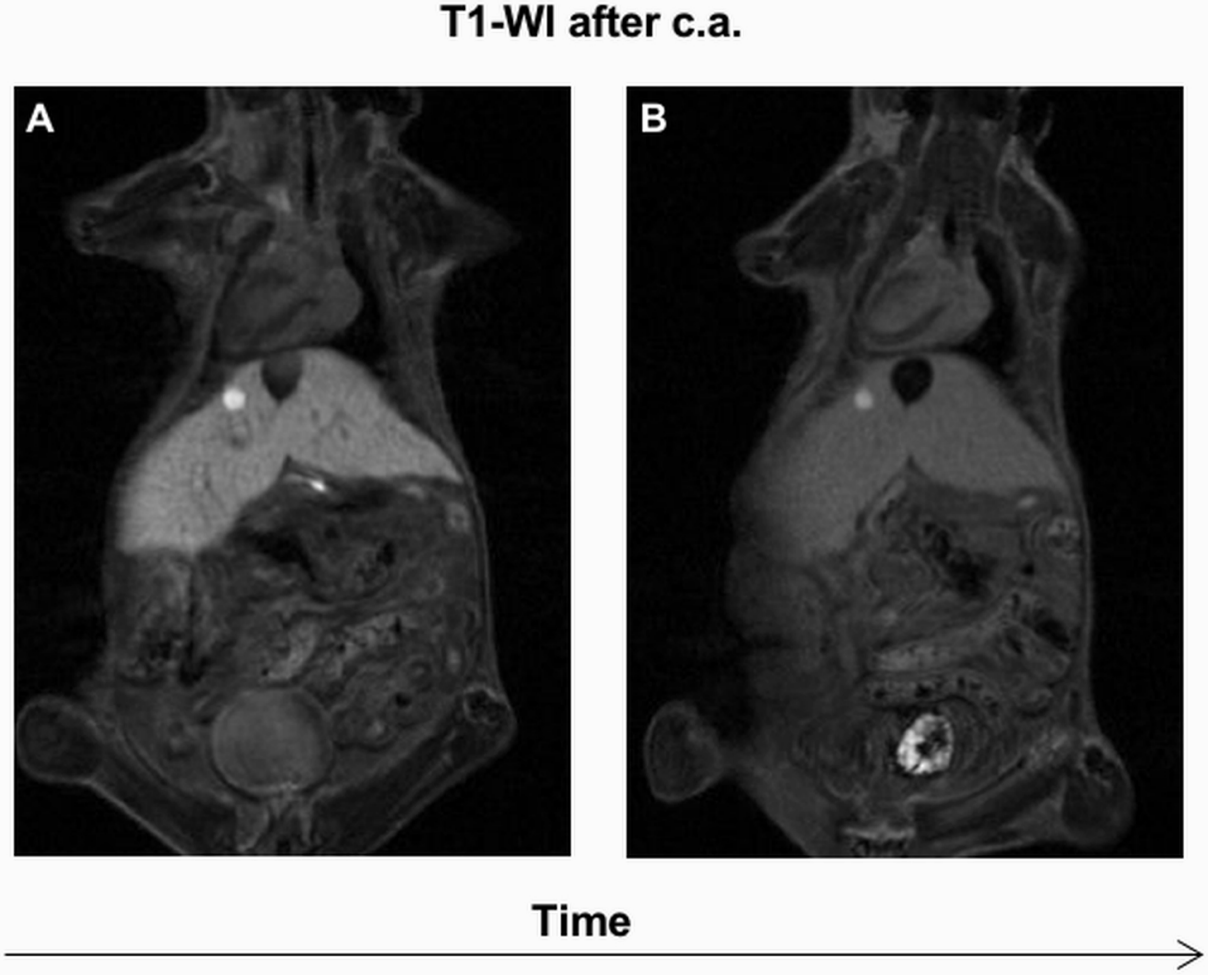
**Growth characteristics of IHCA.** Tumor measurement in LS-*G6pc*^−/−^ mice was performed on the basis of T1-weighted contrast-enhanced scans. (A) A hyperintense lesion was detected on T1-WI after c.a. at the first MRI evaluation. (B) One month later, the lesion had not changed in size and was classified as IHCA by immunohistochemistry.

Time-dependent monitoring demonstrated that hyperintense lesions (*n*=4) on contrast-enhanced T1-WI, without evidence of hyperintensity on T2-WI, had a slow growth rate. All these lesions were identified by immunohistochemistry as IHCA. A representative nodule is shown in Fig. 4.

**Fig. 5.**
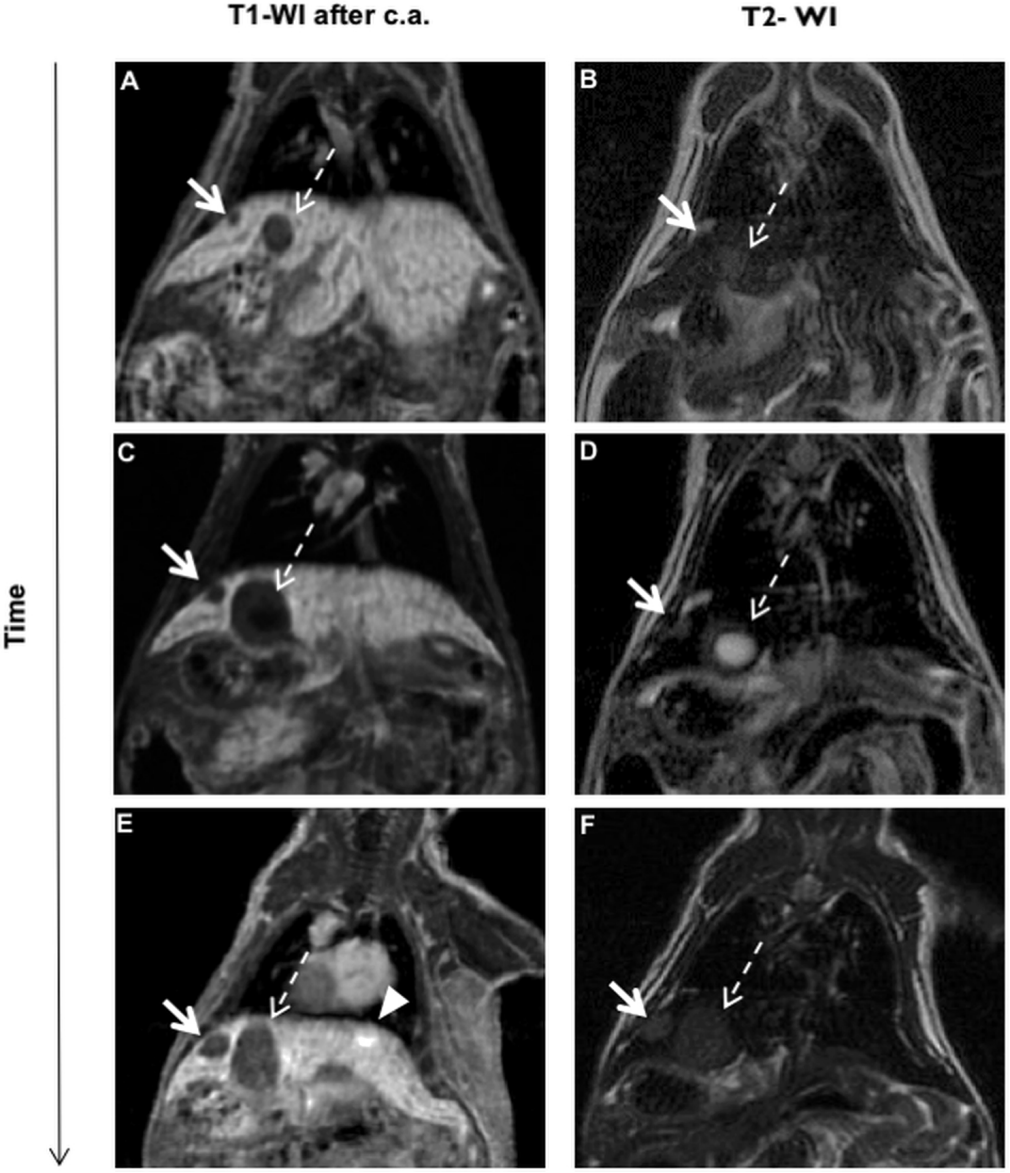
**Time-dependent monitoring of hepatic lesions.** (A-F) LS-*G6pc*^−/−^ mice were monitored every 4 weeks for 3 months, and the tumor growth was analyzed by measuring lesion diameter using the off-line workstation. At the first MRI evaluation (A,B), one mouse showed two lesions that were both hypointense on contrast-enhanced T1-WI but that showed different signals on T2-WI imaging: the small nodule was hypointense (arrows), whereas the bigger nodule was hyperintense (dashed line arrows). After 1 month (C,D), one lesion (arrows) had slightly changed in size and had become hyperintense on T2-WI, while the other lesion (dashed line arrows) had rapidly increased in size and presented with a large central cystic portion that can denote a necrosis area (bright signal intensity). At the third MRI evaluation (E,F), the small lesion (arrows) showed a size increase of 50%, suggesting a high growth rate, while the other lesion (dashed line arrows) had not changed in size. Both lesions were hypointense on T1-WI after c.a. and hyperintense on T2-WI. They were classified by immunohistochemistry as UHCA. A third lesion, hyperintense on contrast-enhanced T1-WI (arrowhead) and isointense on T2-WI, was detected and identified as an IHCA.

At the first MRI evaluation, one of these mice showed two very hypointense nodules on T1-WI after contrast administration (Fig. 5A). Interestingly, these two lesions showed different signals on T2-WI imaging: the smaller one was, at the first MRI examination, isointense (Fig. 5B, arrow), while the bigger one was mildly hyperintense (Fig. 5B, dashed line arrow).

After 1 month, one of the lesions had only slightly changed in size (Fig. 5C, arrow) but had become mildly hyperintense on T2-WI (Fig. 5D, arrow), while the other lesion had significantly increased in size (Fig. 5C, dashed line arrow), remaining hyperintense on T2-WI (Fig. 5D, dashed line arrow) with an internal cystic degeneration.

At the third MRI evaluation, the smaller lesion showed 50% increase in size, suggesting a fast growth rate (Fig. 5E, arrow). Both lesions remained mildly hyperintense on T2-WI (Fig. 5F) and were classified by immunohistochemistry as UHCA.

These findings support the hypothesis that lesions hypointense on hepatobiliary contrast phase (HBP) and mildly hyperintense on T2-WI are rapidly growing tumors. This preliminary experiment underlies the need for further studies to better understand the real biological behavior of UHCA.

A third lesion, hyperintense on post-contrast T1-WI (Fig. 5E, arrowhead) was visualized at this stage and characterized as an IHCA.

## DISCUSSION

Several animal models of liver disease have been developed and extensively utilized in many preclinical medical research studies to investigate disease onset and progression (Fausto and Campbell, 2010; Gabele et al., 2011; Jacobs et al., 2016; He et al., 2015; Yan et al., 2011). Development of HCA in LS-*G6pc*^−/−^ mice is the consequence of a single gene mutation that inhibits G6Pase function and leads to liver degeneration and tumorigenesis. Thus, LS-*G6pc*^−/−^ mice are of major interest because they mimic spontaneous HCA formation with aging and evolution into HCC as in GSD1a patients.

To date there has been a lack of an appropriate *in vivo* detection system that allows continuous monitoring of tumor onset and progression. HCC high-risk patients are monitored through imaging surveillance, and focal nodule characterization is performed with multiphase contrast material-enhanced computed tomography (CT) and/or MRI. Because of variability in lesion interpretation, the Liver Imaging Reporting and Data System (LI-RADS) was created with the aims of improving HCC diagnosis and therapeutic management (Purysko et al., 2012).

Hepatobiliary contrast agents have significantly increased diagnostic accuracy in the detection and characterization of focal liver lesions and its role was recently recognized in the latest version of CT/MRI LI-RADS criteria (v2017), which considers HBP as an ancillary feature in lesion characterization i.e. HBP hypointensity favoring malignancy in general and HBP isointensity favoring benignity (Elsayes et al., 2017).

We set up a protocol to assess the detection level and quantify the longitudinal evolution of liver lesions in LS-*G6pc*^−/−^ mice using non-contrast T1-WI/T2-WI and non-dynamic post-contrast hepatobiliary imaging. We based our differential diagnosis on features considered ancillary by LI-RADS, especially mild to moderate T2 hyperintensity. The hepato-specific contrastographic phase allows the differentiation of lesions on the basis of hepatocyte function and contrast uptake. We used Gd-BOPTA, which, in the clinical practice, has high relaxivity values (Shellock et al., 2006) but only 5% of biliary excretion at 1-2 h after administration. The use of Gd-BOPTA in mice has two main advantages: hepatobiliary phase is already present ∼10 min after contrast administration, and hepatobiliary excretion is ∼50%, because of a different pharmacokinetic profile, as previously demonstrated (Lorusso et al., 1999).

The detection of small HCC is challenging in the clinical practice. In fact, the detection rate of human HCC <1 cm in diameter is only 34% (Lauenstein et al., 2007). Our protocol allows the visualization and monitoring of small HCC/HCA nodules with a diameter of 1 mm and represents an important improvement in imaging technique of murine liver nodules, compared with a study reporting some difficulty in detecting lesions ≤2 mm (Freimuth et al., 2010). The major advantage of our protocol is the possibility to detect high-risk adenoma subtypes. In fact, by combining information on all sequences, and especially contrast-enhanced T1-WI, we were able not only to detect very small size lesions but also to characterize and differentiate them into different histological HCA subtypes.

UHCA remains the less-characterized adenoma subtype, with no specific genetic/pathologic abnormalities and no specific MRI pattern. In fact, UHCA showed all four different contrast enhancement patterns and the majority were isointense on T2-WI. During the follow-up study, we noticed that UHCA showing an MRI pattern typical of IHCA had a slow growth rate, whereas UHCA with evolving features and with an MRI pattern typical of bHCA displayed a high growth rate. Bioulac-Sage and collaborators reported that β-catenin activation was not detected in all nodules of the same patient with adenomatosis, and suggested that β-catenin mutation could occur later during adenoma carcinogenesis, promoting malignant transformation (Bioulac-Sage et al., 2007). Further studies will be required to better understand oncogenesis, and to identify possible differences in clinical features and pathologic and radiologic characteristics of this heterogeneous HCA subtype. In this respect, due to the rarity of UHCA, preclinical models can be useful tools to improve our knowledge in this field. Nevertheless, our results suggest that some UHCA lesions could be considered as the precursors of bHCA at an early stage of carcinogenesis. We did not assess the correlation between tumor markers and high-risk adenomas because tumor markers are most useful for monitoring response to therapy and detecting early relapse. In fact, with the exception of prostate-specific antigen (PSA), tumor markers do not have sufficient sensitivity or specificity for use in screening. Moreover, when proliferating index biomarkers are analyzed, contradictory results are frequently reached, leading to incorrect diagnosis (Rodríguez-Enríquez et al., 2011). Nevertheless, a future study aimed at correlating information on high-risk tumor obtained with MRI analysis and tumor markers may be important to establish an efficient and reliable method to differentiate between high-risk versus low-risk adenomas.

We show here that bHCA lesions display MRI features distinguishable from those exhibited by IHCA or UHCA lesions, indicating that our protocol is a reliable method for the identification of high-risk HCA. This is an important achievement because GSD1a patients often develop multiple adenomas that may originate from independent clones and therefore have different molecular subtypes. Because HCA is a rare disease, the information in the literature to help guide treatment is low. The possibility of characterizing the different HCA subtypes in patients with multiple adenomas is important for planning surveillance and treatment.

In conclusion, this is the first study to report the possibility of differentiating the various subtypes of hepatic adenoma using MRI imaging. In this respect, the establishment of an optimized MRI protocol to detect and analyze liver lesions in our mouse model for GSD1a opens the possibility of applying such a protocol to patients for non-invasive diagnostic purposes. A very recent study utilized a similar MRI protocol to screen for HCC in patients with cirrhosis or chronic hepatitis B. Reported results indicate that MRI characteristics similar to ours are suggestive of malignant transformation (Tillman et al., 2018). We hope that our findings can be translated to the human setting and therefore constitute the basis for the development of an appropriate screening protocol for GSD1a patients.

## MATERIALS AND METHODS

### Housing and breeding of mice

Male and female LS-*G6pc*^−/−^ mice (Resaz et al., 2014), aged 13-18 months, were included in the study. All animals were maintained in a conventional animal facility in 12-h light/dark cycles, fed *ad libitum* and monitored for their lifespan. All animal studies were reviewed and approved by the Ethical Committee for Animal Experimentation (CSEA) as Animal Use Project No. 291, communicated to the Italian Ministry of Health with regard to the article of the D.lgs 116/92, and carried out at the animal facility of the National Institute for Cancer Research (Genova, Italy).

### MRI analysis

MRI was performed with a clinical 3T MR system (Signa EXCITE^®^HDxT, GE Healthcare, Milwaukee, WI, USA). Qualitative and quantitative analyses were performed on a commercially available workstation (AW Volume Share 2, ADW 4.4, GE Healthcare, Milan, Italy).

Mice were anesthetized by intraperitoneal injection of xylazine (30 mg/kg) and ketamine (100 mg/kg) and positioned in a prototype coil (birdcage linear coil, transmit/receive coil, 100 mm long, 55 mm diameter, tuned at 127.6 MH, Flick Engineering Solutions BV&Baio G&GE Milwaukee, WI, USA), placed on a support filled with warm water to preserve mice from hypothermia. For contrast enhancement, 0.05 mmol/kg body weight Gd-BOPTA (MultiHance^®^) was administered by tail vein injection. The room temperature during experiments was 23°C, and the mean acquisition time was limited to 35 min by the spontaneous awakening of mice.

A short localizer sequence was performed and the field of view (FOV) was centered on the upper abdomen. Based on these images, coronal before and after contrast administration, T1-weighted and T2-weighted scans were planned and acquired with parameters in chronological order, as shown in Table 4. Each pre- and post-contrast sequence lasted ∼7 min. The acquisition of all scans lasted ∼25 min. To obtain an HBP, the T1-WI sequences were acquired ∼10 min after contrast administration.

**Table 4.**
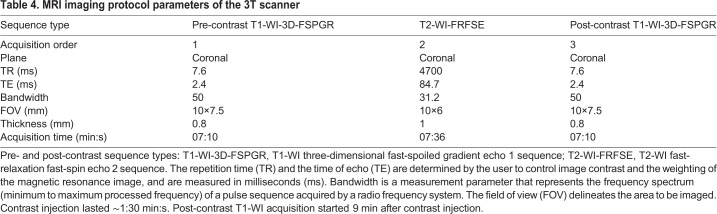
**MRI imaging protocol parameters of the 3T scanner**

A total of 40 animals aged 13-18 months were scanned by MRI and checked for focal lesions in all sequences acquired. Twenty-five mice were scanned once and sacrificed after the MRI session. The remaining 15 mice were monitored every 4 weeks for 3 months to evaluate lesion growth and complication onset, such as rupture and bleeding (see Table 2).

We performed both quantitative and qualitative analyses. We applied a qualitative analysis using a five-point scale to T1-WI and T2-WI; the reader assessed the signal intensity (SI) of the representative liver lesion relative to the adjacent liver parenchyma: 1, very hypointense; 2, mildly hypointense; 3, isointense; 4, mildly hyperintense; and 5, very hyperintense (as biliary cysts) (Ngu et al., 2016). We defined hypointense lesions as those that were unequivocally darker than surrounding parenchyma, hyperintense lesions as those that were unequivocally brighter than surrounding parenchyma and isointense lesions as those that were not distinguishable from surrounding parenchyma.

Quantitative analysis was expressed as relative signal intensity ratio (RelSI) for each lesion, calculated both on T1-WI after contrast and on T2-WI. Lesion and liver SI was measured using an operator-defined region of interest (ROI). After defining the ROIs, we calculated the RelSI according to the formula: RelSI=SI lesion/SI liver.

On T1-WI after contrast, we considered hypointense lesions as those that were with a RelSI ≤0.8 and hyperintense lesions as those that were with a RelSI ≥1.2. As isointense lesions were not distinguishable from the surrounding parenchyma, they were not measurable (Table 1). Mildly hyperintense lesions on T2-WI had a RelSI between 1.4 and 3.3, unequivocally distinguishable from very hyperintense lesions (like cysts) that showed a RelSI of 12-13.

Imaging acquired after contrast administration allowed the clear detection of hepatic lesions and the distinguishing of four different patterns of contrast uptake: isointense, very hyperintense and very hypointense lesions, and lesions with a peripheral contrast enhancement (Fig. 3A-E).

These experiments demonstrated that the optimized parameters shown in Table 4 allow precise/high spatial resolution MRI of small murine liver tumors. In fact, 18 of 40 animals (45%) showed development of focal lesions, ranging between 1 mm and 10 mm in diameter, with different combinations of MRI characteristics and mainly detectable on contrast-enhanced T1-WI. We observed a total number of 41 liver lesions (Tables 1 and 3).

### Histology and immunohistochemistry

Mice were sacrificed shortly after the last MRI acquisition. All livers were macroscopically analyzed for focal hepatic nodules. Before liver sampling, the orientation of all liver lobes, relative to the longitudinal, transversal and sagittal axis of the animal, was marked, and the size and location of the macroscopic tumor nodes were measured with a caliper and correlated with the MRI findings. Part of the respective liver was then embedded in paraffin and used for diagnostic procedures. The rest of the liver was frozen for further analysis.

All tissue samples were fixed in 10% buffered formalin for 24 h and paraffin embedded. For histological analysis, 4-μm-thick serial sections were stained with Hematoxylin and Eosin. Reticulin was visualized by Gomori staining. Immunostaining was performed with anti-GS (Zulehner et al., 2010) (1:500; ab49873, Abcam, Cambridge, UK) and anti-SAA (1:100; PAB795Mu01, Cloud-Clone, Houston, TX, USA) antibodies. Reactions were developed using an EnVision^+^ System-HRP (DAB) (DAKO, Carpinteria, CA, USA). After immunostaining, slides were counterstained with Hematoxylin. Positive and negative controls were included for each run.

## References

[DMM038026C1] Bioulac-Sage P., Rebouissou S., Thomas C., Blanc J.-F., Saric J., Sa Cunha A., Rullier A., Cubel G., Couchy G., Imbeaud S. et al. (2007). Hepatocellular adenoma subtype classification using molecular markers and immunohistochemistry. *Hepatology* 46, 740-748. 10.1002/hep.21743 17663417

[DMM038026C2] Bioulac-Sage P., Balabaud C. and Zucman-Rossi J. (2010). Subtype classification of hepatocellular adenoma. *Dig. Surg.* 27, 39-45. 10.1159/000268406 20357450

[DMM038026C3] Calderaro J., Labrune P., Morcrette G., Rebouissou S., Franco D., Prévot S., Quaglia A., Bedossa P., Libbrecht L., Terracciano L. et al. (2013). Molecular characterization of hepatocellular adenomas developed in patients with glycogen storage disease type I. *J. Hepatol.* 58, 350-357. 10.1016/j.jhep.2012.09.030 23046672

[DMM038026C4] Chou J. Y., Jun H. S. and Mansfield B. C. (2010). Glycogen storage disease type I and G6Pase-beta deficiency: etiology and therapy. *Nat. Rev. Endocrinol.* 6, 676-688. 10.1038/nrendo.2010.189 20975743 PMC4178929

[DMM038026C5] Elsayes K. M., Hooker J. C., Agrons M. M., Kielar A. Z., Tang A., Fowler K. J., Chernyak V., Bashir M. R., Kono Y., Do R. K. et al. (2017). 2017 version of LI-RADS for CT and MR imaging: an update. *Radiographics* 37, 1994-2017. 10.1148/rg.2017170098 29131761

[DMM038026C6] Fausto N. and Campbell J. S. (2010). Mouse models of hepatocellular carcinoma. *Semin. Liver Dis.* 30, 87-98. 10.1055/s-0030-1247135 20175036

[DMM038026C7] Franco L. M., Krishnamurthy V., Bali D., Weinstein D. A., Arn P., Clary B., Boney A., Sullivan J., Frush D. P., Chen Y.-T. et al. (2005). Hepatocellular carcinoma in glycogen storage disease type Ia: a case series. *J. Inherit. Metab. Dis.* 28, 153-162. 10.1007/s10545-005-7500-2 15877204

[DMM038026C8] Freimuth J., Gassler N., Moro N., Günther R. W., Trautwein C., Liedtke C. and Krombach G. A. (2010). Application of magnetic resonance imaging in transgenic and chemical mouse models of hepatocellular carcinoma. *Mol. Cancer.* 9, 94 10.1186/1476-4598-9-94 20429921 PMC2868806

[DMM038026C9] Gabele E., Dostert K., Dorn C., Patsenker E., Stickel F. and Hellerbrand C. (2011). A new model of interactive effects of alcohol and high-fat diet on hepatic fibrosis. *Alcohol Clin. Exp. Res.* 35, 1361-1367. 10.1111/j.1530-0277.2011.01472.x 21463337

[DMM038026C10] He L., Tian D. A., Li P. Y. and He X.-X. (2015). Mouse models of liver cancer: progress and recommendations. *Oncotarget* 6, 23306-23322. 10.18632/oncotarget.4202 26259234 PMC4695120

[DMM038026C11] Jacobs A., Warda A.-S. and Verbeek J. (2016). An overview of mouse models of nonalcoholic steatohepatitis: from past to present. *Curr. Protoc. Mouse Biol.* 6, 185-200. 10.1002/cpmo.3 27248434

[DMM038026C12] Labrune P., Trioche P., Duvaltier I., Chevalier P. and Odièvre M. (1997). Hepatocellular adenomas in glycogen storage disease type I and III: a series of 43 patients and review of the literature. *J. Pediatr. Gastroenterol. Nutr.* 24, 276-279. 10.1097/00005176-199703000-00008 9138172

[DMM038026C13] Lauenstein T. C., Salman K., Morreira R., Heffron T., Spivey J. R., Martinez E., Sharma P. and Martin D. R. (2007). Gadolinium-enhanced MRI for tumor surveillance before liver transplantation: center-based experience. *Am. J. Roentgenol.* 189, 663-670. 10.2214/AJR.07.2171 17715115

[DMM038026C14] Laumonier H., Bioulac-Sage P., Laurent C., Zucman-Rossi J., Balabaud C. and Trillaud H. (2008). Hepatocellular adenomas: magnetic resonance imaging features as a function of molecular pathological classification. *Hepatology* 48, 808-818. 10.1002/hep.22417 18688875

[DMM038026C15] Lee P. J. (2002). Glycogen storage disease type I: pathophysiology of liver adenomas. *Eur. J. Pediatr.* 161 Suppl. 1, S46-S49. 10.1007/BF02679993 12373570

[DMM038026C16] Lei K. J., Shelly L. L., Pan C. J., Sidbury J. and Chou J. (1993). Mutations in the glucose-6-phosphatase gene that cause glycogen storage disease type 1a. *Science* 262, 580-583. 10.1126/science.8211187 8211187

[DMM038026C17] Lorusso V., Arbughi T., Tirone P. and de Haën C. (1999). Pharmacokinetics and tissue distribution in animals of gadobenate ion, the magnetic resonance imaging contrast enhancing component of gadobenate dimeglumine 0.5 M solution for injection (MultiHance). *J. Comput. Assist. Tomogr.* 23 Suppl. 1, S181-S194. 10.1097/00004728-199911001-00023 10608414

[DMM038026C18] Nault J. C., Couchy G., Balabaud C., Morcrette G., Caruso S., Blanc J.-F., Bacq Y., Calderaro J., Paradis V., Ramos J. et al. (2017). Molecular classification of hepatocellular adenoma associates with risk factors, bleeding, and malignant transformation. *Gastroenterology* 152, 880-894. 10.1053/j.gastro.2016.11.042 27939373

[DMM038026C19] Ngu S., Lebron-Zapata L., Pomeranz C., Katz S., Gerst S., Zheng J., Moskowitz C. and Do R. K. G. (2016). Inter-observer agreement on the assessment of relative liver lesion signal intensity on hepatobiliary phase imaging with gadoxetate (Gd-EOB-DTPA). *Abdom. Radiol. (NY)* 41, 50-55. 10.1007/s00261-015-0609-3 26830611 PMC4740974

[DMM038026C20] Oliva M. R. and Saini S. (2004). Liver cancer imaging: role of CT, MRI, US and PET. *Cancer Imaging* 4, S42-S46. 10.1102/1470-7330.2004.0011 18215974 PMC1435346

[DMM038026C21] Purysko A. S., Remer E. M., Coppa C. P., Leão Filho H. M., Thupili C. R. and Veniero J. C. (2012). LI-RADS: a case-based review of the new categorization of liver findings in patients with end-stage liver disease. *Radiographics* 32, 1977-1995. 10.1148/rg.327125026 23150853

[DMM038026C22] Reddy S. K., Kishnani P. S., Sullivan J. A., Koeberl D. D., Desai D. M., Skinner M. A., Rice H. E. and Clary B. M.(2007). Resection of hepatocellular adenoma in patients with glycogen storage disease type Ia. *J. Hepatol.* 47, 658-663. 10.1016/j.jhep.2007.05.012 17637480

[DMM038026C23] Resaz R., Vanni C., Segalerba D., Sementa A. R., Mastracci L., Grillo F., Murgia D., Bosco M. C., Chou J. Y., Barbieri O. et al. (2014). Development of hepatocellular adenomas and carcinomas in mice with liver-specific G6Pase-alpha deficiency. *Dis. Model. Mech.* 7, 1083-1091. 10.1242/dmm.014878 25147298 PMC4142728

[DMM038026C24] Rodríguez-Enríquez S., Pacheco-Velázquez S. C., Gallardo-Pérez J. C., Marí­Hernández A., Aguilar-Ponce J. L., Ruiz-García E., Ruizgodoy-Rivera L. M., Meneses-García A. and Moreno-Sánchez R. (2011). Multi-biomarker pattern for tumor identification and prognosis. *J. Cell. Biochem.* 112, 2703-2715. 10.1002/jcb.23224 21678471

[DMM038026C25] Shellock F. G., Parker J. R., Pirovano G., Shen N., Venetianer C., Kirchin M. A. and Spinazzi A. (2006). Safety characteristics of gadobenate dimeglumine: clinical experience from intra- and interindividual comparison studies with gadopentetate dimeglumine. *J. Magn. Reson. Imaging.* 24, 1378-1385. 10.1002/jmri.20764 17078095

[DMM038026C26] Stoot J. H. M. B., Coelen R. J. S., De Jong M. C. and Dejong C. H. C. (2010). Malignant transformation of hepatocellular adenomas into hepatocellular carcinomas: a systematic review including more than 1600 adenoma cases. *HPB (Oxford)* 12, 509-522. 10.1111/j.1477-2574.2010.00222.x 20887318 PMC2997656

[DMM038026C27] Tillman B. G., Gorman J. D., Hru J. M., Lee M. H., King M. C., Sirlin C. B. and Marks R. M. (2018). Diagnostic per-lesion performance of a simulated gadoxetate disodium-enhanced abbreviated MRI protocol for hepatocellular carcinoma screening. *Clin. Radiol.* 73, 485-493. 10.1016/j.crad.2017.11.013 29246586

[DMM038026C28] Van Aalten S. M., Thomeer M. G., Terkivatan T., Dwarkasing R. S., Verheij J., De Man R. A. and Ijzermans J. N. M. (2011). Hepatocellular adenomas: correlation of MR imaging findings with pathologic subtype classification. *Radiology* 261, 172-181. 10.1148/radiol.11110023 21875850

[DMM038026C29] Yan A. W., Fouts D. E., Brandl J., Stärkel P., Torralba M., Schott E., Tsukamoto H., Nelson K. E., Brenner D. A. and Schnabl B. (2011). Enteric dysbiosis associated with a mouse model of alcoholic liver disease. *Hepatology* 53, 96-105. 10.1002/hep.24018 21254165 PMC3059122

[DMM038026C30] Yoneda N., Matsui O., Kitao A., Kozaka K., Gabata T., Sasaki M., Nakanuma Y., Murata K. and Tani T. (2012). Beta-catenin-activated hepatocellular adenoma showing hyperintensity on hepatobiliary-phase gadoxetic-enhanced magnetic resonance imaging and overexpression of OATP8. *Jpn. J. Radiol.* 30, 777-782. 10.1007/s11604-012-0115-2 22911100

[DMM038026C31] Zucman-Rossi J., Jeannot E., Nhieu J. T., Scoazec J.-Y., Guettier C., Rebouissou S., Bacq Y., Leteurtre E., Paradis V., Michalak S. et al. (2006). Genotype-phenotype correlation in hepatocellular adenoma: new classification and relationship with HCC. *Hepatology* 43, 515-524. 10.1002/hep.21068 16496320

[DMM038026C32] Zulehner G., Mikula M., Schneller D., van Zijl F., Huber H., Sieghart W., Grasl-Kraupp B., Waldhör T., Peck-Radosavljevic M., Beug H. et al. (2010). Nuclear beta-catenin induces an early liver progenitor phenotype in hepatocellular carcinoma and promotes tumor recurrence. *Am. J. Pathol.* 176, 472-481. 10.2353/ajpath.2010.090300 20008139 PMC2797905

